# Nonsyndromic Mandibular Symphysis Cleft

**DOI:** 10.1155/2014/682163

**Published:** 2014-03-03

**Authors:** Leela Krishna Guttikonda, Koteswara Rao Nadella, Vijayalakshmi Uppaluru, Rama Mohan Kodali, Ranganadh Nallamothu

**Affiliations:** Department of Oral & Maxillofacial Surgery, Drs. S&NR SIDS, Chinaoutpalli, Krishna District, Andhra Pradesh, Gannavaram 521286, India

## Abstract

Median cleft of lower lip and mandible is a rare congenital anomaly described as cleft number 30 of Tessier's classification. In minor forms only lower lip cleft is seen. We report the case of a patient with median cleft of lower lip, severe ankyloglossia, cleft of mandibular symphysis, and residual cleft involving on right soft palate and associated with other facial clefts. These deformities were corrected in multiple stage procedure, consisting of release of the tongue from floor of the mouth and lower alveolus and fixation of the mandibular cleft done with right iliac bone graft using stainless steel miniplate.

## 1. Introduction

Orofacial clefts are the most common facial malformations in all populations and ethnic groups. Tessier cleft number 30 is a rare congenital deformity and presents with varying degree of severity from isolated median cleft of the lower lip to cleft of the manubrium sterni involving the mandible, tongue, floor of the mouth, hyoid bone, thyroid cartilage, and strap muscles of the neck; the cleft is frequently associated with ankyloglossia and median web in the neck extending from chin, causing neck contracture [1]. Couronne' reported the first account of this anomaly in 1819. Since then very few cases have been reported in the literature with different variations. We came across a patient with multiple facial clefts associated with mandibular symphysis cleft and ankyloglossia [2].

## 2. Case Report

**Figure 1:**
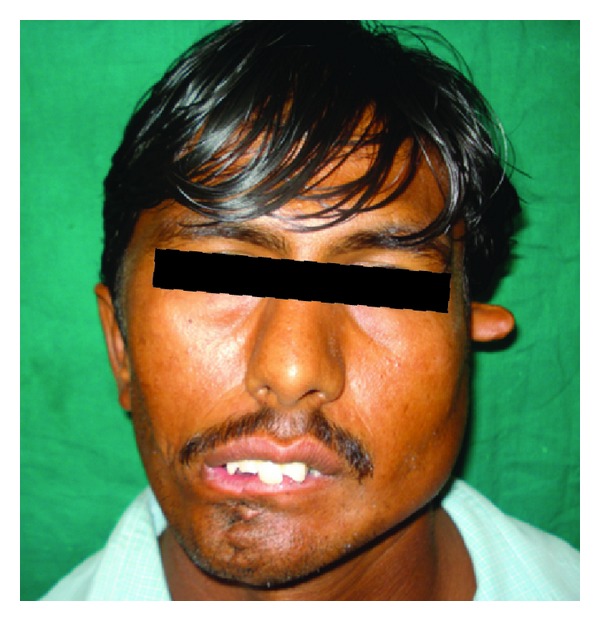
Frontal view.

**Figure 2:**
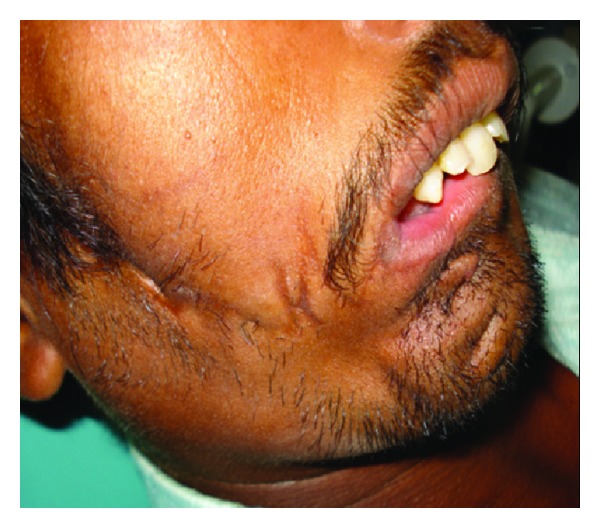
Right lateral view.

**Figure 3:**
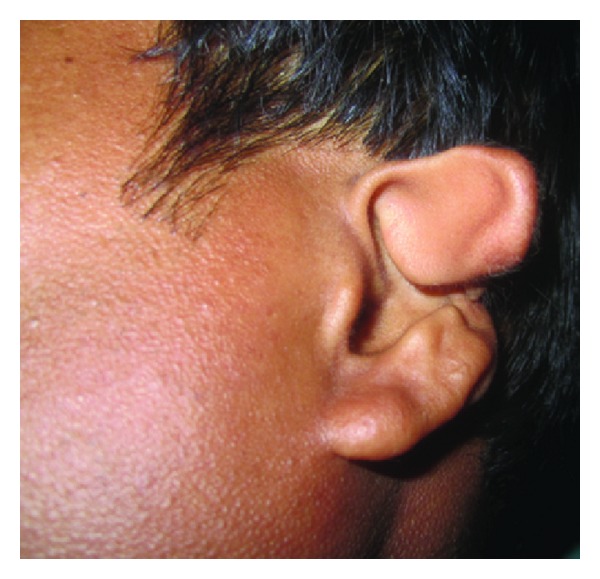
Left ear cleft.

A 28-year-old male patient (Figure 1) reported to our unit with chief compliant of difficulty in speech, disfigurement of face, and difficulty in mastication of food since his childhood. Patient underwent surgery for closure of facial clefts at the age of 1 month under General anaesthesia and at the age of 16 years. On extraoral clinical examination gross facial asymmetry noticed with mandibular midline deviation towards right side, upper, and lower lips was incompetent and previously operated extraoral scars were noticed extending from right side of the corner of mouth, cheek, and auricle and scar on midline of lower lip (Figure 2) and noticed nonoperated clefts on left ear (Figure 3).

**Figure 4:**
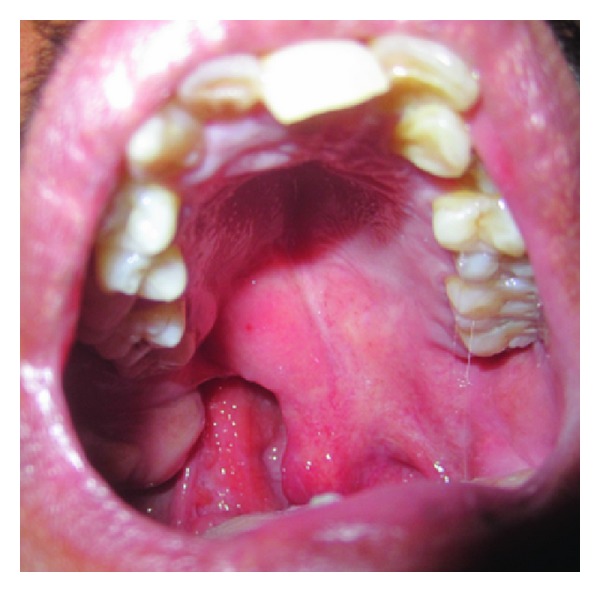
Residual cleft on right soft palate.

**Figure 5:**
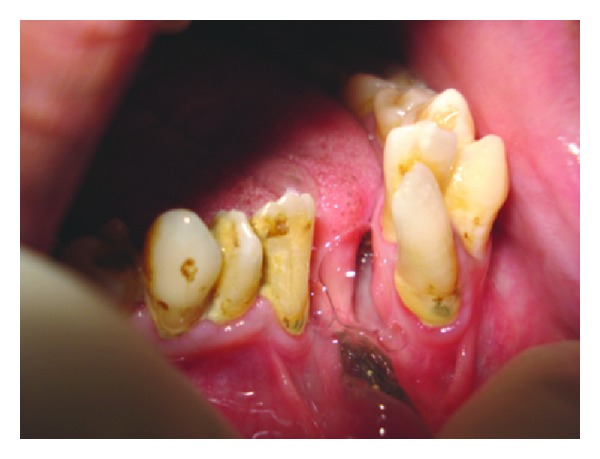
Ankyloglossia.

**Figure 6:**
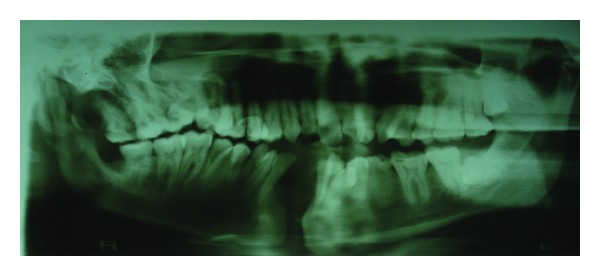
Preoperative orthopantomogram shows mandibular symphysis cleft.

On intraorally soft tissue examination scar was noticed on right side of the hard palate showing with a residual cleft on right soft palate (Figure 4). On hard tissue examination mandibular symphysis cleft with ankyloglossia (Figure 5) was noticed and on bimanual palpation 2 halves of mandibular segments were freely movable. Midline shift towards right side and crowding in the lower anteriors, supernumerary teeth distal to 18 and between 44 and 45 region, retained deciduous teeth of 83 were noticed and missing 43. On Radiographic examination (Figure 6) mandible symphysis cleft was noticed between 31 and 41 and impacted 3rd molars of 28 and 38.

**Figure 7:**
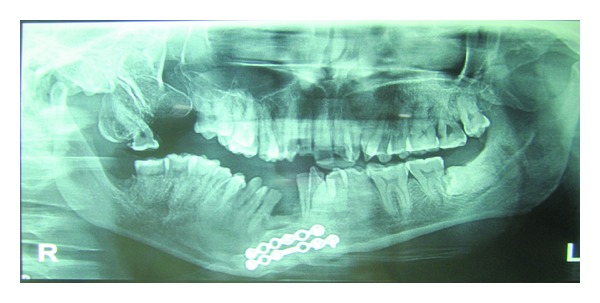
Postoperative orthopantomogram.

**Figure 8:**
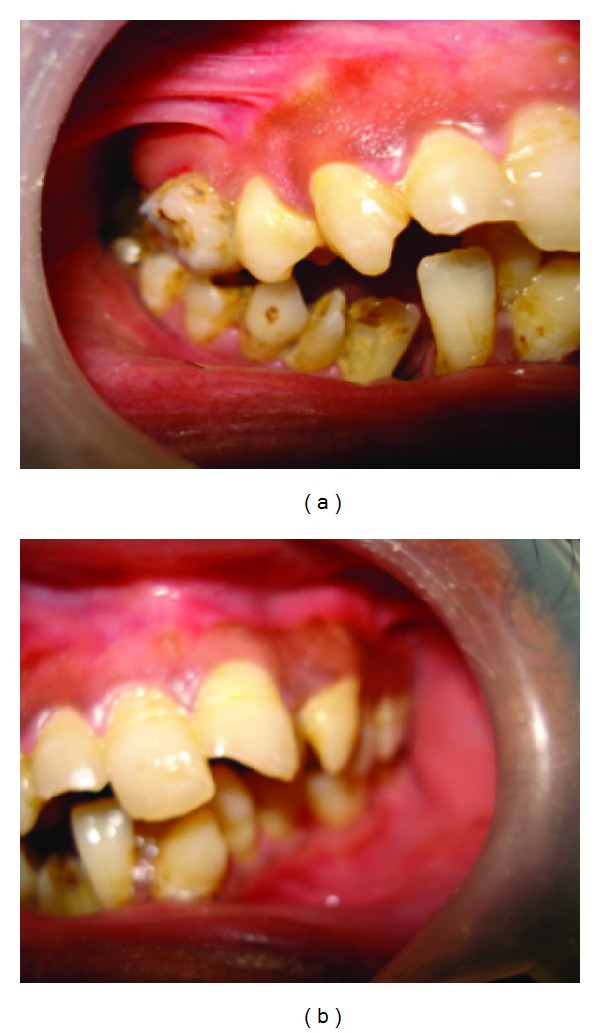
(a) and (b): right and left lateral occlusal views.

Under general anaesthesia, routine preparation of patient was done and throat pack was placed; IMF done from 6/6 to 6/6 with lower segmental arch bar was placed guiding the occlusion. The tongue was released, and a frenectomy of the lingual frenulum was performed after the frenectomy; an incision was made in the mandibular buccal sulcus between the cuspids; and a mucoperiosteal flap was raised; the cleft bony margins were exposed on either sides till the lower border of mandible, genioglossus, and hyoid muscles were released from mucosal part of lower lip region. Iliac crest bone graft was taken from right iliac region and contoured to the symphysis of the mandible using 2 mm 6 hole; with gap and without gap plates were placed in both superior and inferior borders. Donor site was sutured with 3-0 vicryl and dynplaster dressing was given to reduce hematoma formation. Recipient site that is intraoral symphysis region was sutured after undermining the margins for removal of fibrous tissue and was sutured with 3-0 and 2-0 vicryl. Postoperative radiograph (Figure 7) was taken immediately and after 6 months of surgery, the bone graft was well integrated with full union of the mandible. The tongue presents with normal movements. Occlusion is satisfactory. (Figures 8(a) and 8(b)).

## 3. Discussion

Orofacial clefts are congenital structural anomalies that affect 1/1000 live births. Their frequent occurrence as well as their extensive psychological, surgical, speech, and dental involvement emphasize the importance of understanding the underlying causes [3]. The etiology of orofacial clefts is complex, including multiple genetic and environmental factors. A variety of congenital syndromes affecting the face occur due to defects involving the first and second branchial arches [4].

Embryologically, the mandible develops from the cartilage of the first pharyngeal arch, the mandibular process, known as Meckel's cartilage. The mandible is derived from ossification of an osteogenic membrane formed from ectomesenchymal condensation at 36 to 38 days of development. In the mental region, on either side of the symphysis, 1 or 2 small cartilages appear, and endochondral ossification commences in the seventh month in utero to form a number of mental ossicles. These ossicles become incorporated into the intramembranous bone when the symphysis menti is converted from a syndesmosis to a synostosis during the first postnatal year. This failure may be a deficiency or delayed growth of the mesenchyme. Also, considering the developmental stage of the organs involved, it appears that the disturbing influence occurs at the end of the fifth or at the beginning of the sixth week of gestation [5].

Developmental anomalies of structures derived from the upper half of the first branchial arch are common, giving rise to deformities such as cleft lip or cleft palate. However, abnormal or incomplete development of structures derived from the lower half of the first branchial arch is rare due to a failure of fusion of the first pair of branchial arches or a failure of mesodermal penetration into the midline of mandibular part of the first branchial arch. This failure may be a deficiency or delayed growth of the mesenchyme. This may present as a complete or incomplete cleft of the lower lip, mandible, and tongue with occasional associated deformities of soft tissue structures in the neck derived from the lower branchial arch. [6]

The most common abnormalities related to this condition are congenital heart disease, absence of the hyoid bone, and neck contracture. Facial clefts could be in general part of syndromes. However, in the presented case, we could not identify a specific syndrome.

In 1971, Millard et al. [7] thought that the failure of the mandibular processes to fuse may keep the ventral ends of the succeeding arches from uniting in as much as fusion proceeds from above. This may explain the absence of the hyoid bone, thyroid cartilage, strap muscles, and manubrium in some of the more severe cases.

In 2002, Almeida et al. [6] reported the great variation in the severity of midline clefts of the lower lip and mandible and their association with other congenital defects, which are caused by external factors that can result in widespread disturbances in embryologic development. This theory, however, can be easily excluded because of the fact that localization of the clefting deformity of the mandible involves the midline in all cases described.

In 2004, Rana et al. [8] proposed that there is only one branchial arch during early embryonic period (7th week) into which two mandibular processes grows with a groove in midline. Hypoplasia of the mandibular processes during early embryonic period will lead to severest cleft of the mandible extending into the neck. During the late embryonic period less severe median clefts will develop.

Johnson et al. also proposed that disturbances of the outgrowth of bone centres of the mandible result in mandibular and soft tissue clefting, whereas defects in the merging process produce a notch of the vermilion or a higher cleft of the lower lip with or without involvement of the alveolar process of the mandible. Radiographic evaluation of craniofacial deformities is necessary to define aberrant anatomy, plan surgical procedures, and evaluate the effects of craniofacial growth and surgical reconstructions [4]. Based on this background, we noticed similar defects in our presented case.

### 3.1. According to American Association of Cleft Palate Rehabilitation Classification (AACPR)

The classification suggested by Harkins and associates (1962) and endorsed by the American Association of Cleft Palate Rehabilitation Classification (AACPR) is based on the same principles used by Kernahan and Stark.

- Cleft of primary palate.
  - Cleft lip, unilateral, bilateral, median, prolabium, and congenital scar.
  - Alveolar cleft, unilateral, bilateral, and median.
- Cleft of palate proper.
  - Involving soft palate.
  - Involving hard palate.
- Mandibular process cleft.
  - Mandibular cleft lip.
  - Mandibular cleft.
  - Lower lip pits.
- Nasoocular cleft, extending from narial region to the medial canthal region.
- Oroocular cleft, extending from the angle of the mouth towards the palpebral fissure.
- Oroaural cleft, extending from the angle of the mouth towards the ear.

Concerning treatment, the rarity and variation of severity of the condition are responsible for the lack of a consensus on the nature and timing of the corrective surgery. The majority of the authors propose correction of the soft tissue structures as soon as possible, so as not to cause feeding and/or speaking problems and mandibular bone grafting in later stages of life. The timing of treatment of mandibular clefts by most surgeons is when the child is about 8 to 10 years to avoid damaging developing tooth buds. However, Sherman and Goulian described the successful management of a complete cleft of the lower lip and mandible in a 1-stage procedure in a 20-month-old child [6].

Armstrong and Waterhouse have suggested that reconstruction should be done after the age of 10 years to avoid damaging developing tooth buds. To simplify, if there is no gap between the two mandibular halves, the second surgery can be deferred till 10 years. While if the patient has feeding or breathing difficulty and the mandibular segments are mobile, an early attempt should be made to stabilise the mandible with bone graft or reconstruction plate.

In our patient, we corrected midline cleft of mandibular symphysis with right iliac crest bone graft and semirigid fixation and also corrected ankyloglossia in a 1-stage procedure in consideration of the age and the stage of development of the patient.
